# “Oh, Another Overdose, for the Love of Pete”: First Responder Perspectives on Overdose Response Technology

**DOI:** 10.5811/westjem.18471

**Published:** 2025-02-12

**Authors:** William Rioux, Stephanie Jones, S. Monty Ghosh

**Affiliations:** * University of Alberta, Faculty of Medicine & Dentistry, Department of Medicine, Edmonton, Alberta, Canada; † Three Hive Consulting, Vancouver, British Columbia, Canada; ‡ University of Alberta, Faculty of Medicine & Dentistry, Department of Internal Medicine, Edmonton, Alberta, Canada

## Abstract

**Background:**

Overdose response applications and hotlines are novel overdose response technologies (ORT)/virtual harm reduction strategies that have recently emerged as a strategy to reduce the harms associated with the ongoing opioid epidemic. First responders are often the first point of contact for people who have overdosed and play a significant role in responses enacted by these services. In this study our aim was to explore the attitudes and perceptions of first responders on these novel technologies.

**Methods:**

We recruited 17 participants using purposive sampling through the province of Alberta between February–April 2023 including 11 paramedics, two firefighters, and five emergency communications operators. To be included in the study, participants were required to be older than 18 years of age, have the ability to communicate effectively in English, provide verbal informed consent, and work in an emergency responder role. Semi-structured interviews were conducted by two evaluators. When reviewing interview transcripts we used thematic analysis to identify key themes and subthemes.

**Results:**

Participants discussed their current operating procedures, their current perspectives on overdose response hotlines and apps, how they would best integrate them into their current workloads, and how to raise awareness of these services within first-responder communities. Participants were apprehensive about the integration of these services into their current workloads, including their potential benefits, and raised concerns about their efficacy within communities of people who use drugs. Key strategies were raised for the successful integration of these services into emergency responses including providing information to clients and the feasibility of overdose responses by the general public.

**Conclusion:**

This study’s results add to the existing literature on the toll of the overdose epidemic seen within first-response communities. Furthermore, we explored the communities’ diverse perspectives on these novel technologies, including support and concerns, and propose additional strategies for their integration into emergency responses.

## BACKGROUND

Canada continues to face disproportionate rates of mortality resulting from an increasingly toxic drug supply and substance use harms associated with isolation measures put in place during the pandemic, with this region seeing nearly double the number of illicit drug deaths during that time period. 1 More recently, the province of Alberta has seen a dramatic rise in opioid-related emergency service calls per week, which have doubled between January and April 2023, 2 highlighting the need for effective and novel strategies to address this crisis. On the frontlines of this epidemic are first responders, including emergency medical service (EMS) workers, firefighters, and 9-1-1 dispatch operators, who face significantly increased workloads and greater numbers of interactions with people who use drugs (PWUD).

Various harm reduction measures have helped to curb the rates of EMS interactions within this population, 3 yet these frontline workers continue to face increased interactions with PWUD. 2 While not generalizable to the entire nation, first responders in Canada have been cited to have favourable attitudes to the integration of harm reduction in their communities. 4 Often, however, these harm reduction measures are centralized within large urban centers, with rural communities experiencing higher rates of fatal overdose. 5 One novel measure that has emerged to both counteract the negative impacts of isolation and to provide more equitable access to rural communities has been the advent and scaling of novel, virtual harm reduction services such as overdose response applications and hotlines, sometimes referred to in the literature as mobile overdose response services. 6–8

In particular, services that can be accessed by anyone with a mobile/cell phone, including overdose response hotlines (Canada’s National Overdose Response Service, America’s Never Use Alone, or Massachusetts Overdose Prevention Line) 9,10 and overdose response applications (Digital Overdose Response Service [DORS] app, Lifeguard, and UnityPhilly), 11,12 which allow for the spread of harm reduction beyond their brick-and-mortar counterparts. The former, overdose response hotlines, traditionally connect people using substances to a live operator who will co-create an emergency response plan with an individual using substances and enact said plan should an individual become unresponsive. Overdose response applications, on the other hand, use a smartphone, timer-based system in which people using substances alongside the app are prompted to refresh a timer at regular intervals, followed by connecting them with emergency communications officers should they be unresponsive, and dispatching EMS to their location. 13

As these technologies emerge, it is imperative to understand the perspectives of stakeholders who are most impacted by these types of services. Indeed, while the current literature suggests that these services are acceptable for many PWUD, 14–16 in particular gender minorities, 17 there is a dearth of literature on the perspectives of various first responders. 18 However, interactions between PWUD and EMS present points of critical intervention and valuable interactions with the healthcare system as many individuals choose not to seek higher levels of care, potentially arising from stigma or precipitated withdrawal resulting from high doses of naloxone administration. 19,20 A recent review by Bolster et al highlights the urgent need for addressing the “overlooked and under-researched” role of EMS and other first responders in the care of PWUD. Indeed as highlighted within their review, first responders are in an excellent position to provide additional support and resources to reduce the harms associated with substance use. 21 In our study we aimed to determine the perceptions of first responders on novel overdose response applications and hotlines including their current knowledge of and future potential to integrate these services into their scope of practice.

**Population Health Research Capsule**

What do we already know about this issue?

*Overdose response technologies (ORT) aim to address the rising mortality from the opioid crisis; however, no research examines first-responder perspectives of ORT.*

What was the research question?

*What are first responders’ perceptions of ORT and their integration into current scope of practice?*

What was the major finding of the study?

*While acknowledging ORT benefits such as outreach to geographically isolated individuals, they expressed concerns about personal and client safety during responses.*

How does this improve population health?

*First responders offer a valuable opportunity to disseminate information about ORT to reduce the mortality rate from the opioid epidemic.*

## METHODS

To understand the perspectives of first responders on overdose response hotlines and apps, we conducted 17 semi-structured interviews of firefighters, paramedics, and emergency service dispatchers within the province of Alberta. Interviews were conducted between February–April 2023. Interview participants were identified through a combination of convenience and snowball sampling through existing networks with Alberta Health Services, a province-wide health authority located in Canada. Participants were initially contacted via email and invited to participate in this study. The province of Alberta is the fourth largest province in Canada with a population of 4.8 million as of the most recent estimates (2024) 22 ; this province also sees the second highest rates of fatal overdoses within the country. 1 The population of this province is primarily concentrated within urban areas (93.4%). 23 There have been 200 uses of the Provincial Overdose Response Application DORS since its launch in 2021 until when these interviews were conducted (April 2023). 24 Similarly, the National Overdose Response Service was used 1,108 times across the three prairie provinces (including Alberta) from December 2020–April 2023. 24

Inclusion criteria required participants to be residents of Alberta at the time of consent, ≥18 years of age, able to communicate effectively in English, and to provide informed verbal consent. In addition, participants had to be employed in an emergency response role, which may be impacted by the use of virtual harm reduction tools (such as paramedics, emergency communications officers or firefighters). Due to the relative recency of overdose response applications and hotlines, participants were not required to have previously responded to a call initiated by these services. To minimize potential interview bias, the interviews were conducted by two masters-level trained female evaluators (SJ and SC**)** who are part of a third-party research organization specializing in qualitative research. There was no previously established relationship between evaluators and interview participants; only the interviewer and interviewee were present on the call. Prior to conducting interviews, participants were provided with a brief information package about the various overdose response hotlines and applications available. No honorarium was granted for participant interviews, and no participants dropped out during or after the interviews were completed.

We consulted with both overdose response hotline and application administrators and PWUD before creating the recruitment package, which included a verbal consent form, contact information of the study personnel, a telephone recruitment script, and a letter detailing the research study. An implementation science framework was used to guide the question design. 25 Telephone interviews ranged from 20–60 minutes in length. The third-party transcription service TapeACall was used to record and transcribe the data. No field notes were taken for this analysis, transcripts were not returned to any participants to confirm validity, and no repeat interviews were conducted. We used the consolidated criteria for reporting qualitative research (COREQ) to guide the reporting of the results. The study received ethical approval from the University of Calgary Conjoint Health Research Ethics Board (REB22-0326).

Qualitative information was encoded via thematic analysis to identify themes that could help organize the perceptions and opinions shared by study participants. Interviews were conducted until data saturation was reached, which was defined as the lack of new themes emerging. 26 The transcripts were independently coded by both interviewers using Dedoose (SocioCultural Research Consultants, LLC, Redondo Beach, CA). After coding the first three transcripts, evaluators met to discuss and refine a codebook that was updated in real time based on joint evaluator agreement. Disagreements between evaluators were resolved through consultation with the project manager (KM) and the principal investigator (MG).

## RESULTS

Through our study, we collected the perspectives of 12 emergency medical first responders and five EMS dispatchers. Due to the geographic variation within our subject pool, respondents also varied significantly in the number of overdose responses they attended. Furthermore, participants varied in their previous awareness of ORT, with 12 having a basic understanding of these services and five without prior knowledge. Years of service and ethnicity data were only collected for 14/17 (82.3%) of individuals; however, of these individuals, 11/14 (78.5%) were White and had an average of 11.2 years of service (SD 7.4). Additional demographic details can be found in Table 1.

Four major themes were identified from the interviews: *the current toll of overdose calls on emergency responders; current awareness of overdose response hotlines and opinion that apps should be improved; acceptability of these services;* and *integration of these services into communities and current workloads.* Outside the frequency of overdose calls, there were no significant differences between participants’ perspectives based on rurality and gender. Key themes, subthemes, and points are summarized in Table 2.

### Current Toll of Overdose Calls on Emergency Responders

Despite the varied number of overdose responses enacted by EMS across the province, participants’ views on the impacts of the overdose crisis were relatively similar. In general, participants noted an increasing call volume and stress on already scarce resources.

*Overdose responses are presenting a pretty significant challenge, in my opinion, for our healthcare system. We take a lot of overdose calls….A lot of EMS resources are spent responding to these calls. –* EMS15

Undoubtedly, many first responders faced significant emotional tolls and a cycle of frustration and exhaustion due to repeated attended calls. Many individuals describe that the impact of this crisis has left them *“jaded”* (EMS 13) and demoralized.

*It can be quite demoralizing as a first responder. I’ve worked with people who have given NARCAN and reversed the effects of narcotics twice to the same person on the same shift. –* EMS 04.

### Acceptability of Overdose Response Hotlines and Applications

After hearing a brief description of the services presented as part of the interview guide (Appendix 1) interviewers explored the perspectives of first responders and dispatchers on the use of overdose response hotlines and applications as a part of a comprehensive harm reduction and treatment strategy. While participant perspectives were mixed on the acceptability of these services in their community, some participants described the apprehension among staff members due to the relative novelty of these services, response times, and safety concerns.

*I think it’s going to be a hard sell to first responders… Talking to other people, our concern was how many false alarms are we going to start going to now?... I think if you explain to them how it works … that would probably help … –* EMS 08

Furthermore, we aimed to determine the perspective of individuals on the two types of services (applications or hotlines) that are currently available. In reference to application-based services, one participant stated:

*They’re a critical piece that needs to be implemented and supported, and people need to be aware of it and, you know, educated. –* EMS 01

Participants additionally recognized the potential to provide support for individuals *“isolated in society”* (EMS 09). Individuals who are using alone, in their own homes, or in First Nations, rural and remote communities could see additional benefits, according to participants. While there were some concerns regarding false alarm calls, both EMS workers and call-takers noted that it would not be outside the norm of those seen with current life-alert systems. Additional apprehension was raised with respect to responder safety when accessing a location, technological issues, and the acceptability of these services among PWUD.

Regarding peer-to-peer services described above, participants held favorable attitudes toward these types of services. Indeed, participants highlighted that relationships developed between callers and operators would greatly benefit this type of service.

*Even just something as simple as that lets them know that there is somebody that actually cares… I think that gives people hope. –* EMS 08

*If it didn’t exist, then you would be at the situation we’re at now. Where high volume of calls … And there has to be a better way of managing* (the high volume of calls related to the opioid crisis). *–* EMS 14

Participants additionally highlighted the opportunity of these services to connect individuals to harm reduction and treatment resources at the appropriate times.

*Being able to have that personal connection and have that conversation and enter into a safety contract that is explicitly chosen by the person with substance abuse concerns. And that potentially could facilitate more appropriate long-term interventions and supports. –* EMS 09

Lastly, some first responders viewed these services as enabling and that they would not deter individuals from using substances.

### Integrating Overdose Response Hotlines and Applications into Communities and Current Workloads Is Feasible

Noting many of the strengths of these services previously outlined by interview participants, questions were raised about how these services may be integrated into current EMS and call-taker workloads. There was significant discourse around the appropriateness of providing information to people who have experienced an overdose, primarily due to altered mental states faced by individuals after overdose reversals.

*In my experience when patients wake up they’re not often in the best state of mind so talking to them at that particular point. –* EMS-06

*I do and I have [recommended these services to patients]… I do think that these types of apps and services, or hotlines, do fill an important gap in our current provisions of care, for this type of thing. –* EMS 03

In contrast, many 911 dispatchers noted that while they would be supportive of providing this education there would be challenges to disseminating knowledge of these services due to the limitations of their protocols and scripts.

*For ECOs (Emergency Communications Operators) at the moment, there’s nothing in the protocols that would allow us to actually do that…it would need, like something added in now. That’s not to say it couldn’t be done, that shouldn’t be done. –* EMS 14

Lastly, the concept of contacting members of the public to potentially provide more timely naloxone administration was discussed. Participants held mixed views about the integration of the public to respond to reported overdoses. While some recognized the value in potentially more rapid intervention and reaching out to a support system to intervene, others were concerned about lay responder safety, liability, training for appropriate responses, potential delays in emergency medical dispatch, and mental health and trauma arising from responses. Participants also raised concerns about potential delays in emergency response should community members be contacted first.

*“I don’t know if I’d be completely comfortable with them administering NARCAN to a complete stranger. Some people wake up swinging, some people don’t wake up at all. –* EMS 04

### Current Awareness of Overdose Response Hotlines and Applications Should be Improved

In general, participants who had heard of the services noted that their knowledge was limited. Participants recalled hearing information about these services through various communication channels, including articles, television programming, province-wide newsletters, emails, posters, and presentations, but many acknowledged that they had only heard of the services in name only. Not surprisingly, the government-funded and Alberta-specific DORS app made up most of individuals’ knowledge on this subject.

*I think it’s an app on the phone and it calls somebody – you set it up before you use and then when the app checks it. Basically, it’s a, I think an alarm. And that’s pretty much all I know. –* EMS11

Some participants suggested that continuing education modules, education days, and continual communication about these services as they develop would build a more concrete understanding of the methodology and rationale of overdose-response hotlines and applications. In contrast, others did not see a need to be educated on these services.

## DISCUSSION

This study is the first to explore the perceptions of first responders regarding various facets of implementing overdose-response hotlines and apps. As previously highlighted, participants were recruited from a province that developed and promoted a provincial virtual harm reduction tool, the DORS app, in April 2021, which is directly linked to their emergency medical system. 27 Tailored messaging on naloxone kits has helped to disseminate these services to individuals and spread their awareness. 28 Other national services, including the National Overdose Response Service and Brave app, are also available within the province. 9,29

### Current Toll of Overdose Calls

The recent COVID-19 pandemic and ongoing opioid overdose epidemic have placed significant strain on EMS within the province and Canada as a whole. Within the previous four months, the province of Alberta has seen double the number of EMS responses to opioid-related events per week. 2 As echoed within the results of our study, previous findings describe the taxing nature of overdose responses, particularly for those who overdose multiple times within a day. 30,31 As demonstrated within our results, EMS workers find this work demoralizing and attribute the overdose crisis to occupational burnout. 32 Indeed, a cross-sectional survey of EMS workers in the state of Pennsylvania in the US found a correlation between the number of overdoses workers responded to and rates of depression among service personnel. 33 Part of this stress has been attributed to role conflicts from first responders noting that they felt they “only provided a temporary, sometimes ineffective, solution for PWUD,” 30,31 focusing on resuscitation only.

We suggest that broadening the scope of services provided by first responders to help with harm reduction education and facilitation of treatment may be a potential solution to help alleviate this burden. 30 Throughout our results, some participants identified the opportunities of introducing these services to PWUD as long-term solutions that connect individuals to support and fill an important gap in healthcare. In conjunction with many well-researched strategies for reducing stress and mental health concerns within this population, 34 expanding the purview of EMS personnel and capitalizing on their connection with PWUD should be considered as a potential support to address the mental health impacts of the overdose crisis on service workers.

### Current Awareness of Overdose Response Hotlines and Applications Should be Improved

Throughout our interviews, many participants were aware of overdose-response hotlines and applications available to PWUD; however, most were not familiar enough with them to the extent of recommending them to others. Indeed, increasing awareness of these services as well as public health messaging for PWUD has been discussed in recent literature. 6,28,35–37 One participant familiar with the technology recommended it to PWUD with whom they have interacted, demonstrating that building a greater awareness of these services among EMS personnel may help spread awareness of both overdose response hotlines and applications and additional harm reduction strategies within PWUD.

Interview participants also suggested that diverse methods be employed to educate their colleagues and staff members on these resources. A multipronged approach to education would likely help best disseminate this knowledge among first responders. This work can be undertaken in conjunction with stigma reduction and trauma-informed care initiatives to increase treatment uptake and harm reduction service access. 38,39

### Acceptability of Overdose Response Hotlines and Applications

Our study found mixed perspectives on the acceptability of these services among first responders and diverse opinions on the types of virtual harm-reduction services. While some inherent strengths were discussed, some participants felt these services and other harm-reduction measures were enabling. These persistent attitudes have been previously described across communities of frontline workers, 40–43 and they persist despite evidence of their efficacy in reducing harms from illicit substance use. 44 As previously mentioned, first responders are often the first and sometimes the only interaction with the healthcare system for PWUD, particularly those who refuse care. Sharing knowledge of evidence-based harm reduction approaches, as well as the evidence for the various virtual harm reduction interventions, could help build an understanding of the importance of these services in dealing with opioid overdoses. In this way, stigma reduction education for first responders has demonstrated positive outcomes for PWUD, including treatment outcomes and individual self-esteem. 45

Similar to previous studies on paramedic-attended overdose events, in this study we found that individuals held concerns about their safety when responding to these situations. 31,46 Drug paraphernalia, post-naloxone aggression, and fears of violence have been discussed throughout various EMS perspectives on overdose response. 31,47 Overdose response hotlines and apps should, therefore, encourage service users to appropriately plan for an overdose event similar to those implemented within the National Overdose Response Service to reduce this risk. 48 Education to service users about removing paraphernalia, unlocking the front door, securing pets at home, and having a “hospital to go bag” filled with extra clothes and toiletries in case they have to go to the hospital are all interventions that could improve both the client and EMS professional experience on these lines.

Additionally, concerns about response times were addressed by participants. In the case of overdose and hypoxemic brain injury, rapid response times would be crucial to prevent severe outcomes. While these services may enable more rapid interventions when using drugs alone, future efforts should be considered to educate PWUD on response times within their areas and the resulting greater risks of overdose. In particular, rural areas may see response times nearly double those in more urban jurisdictions. 49 Emergency response plans made in consultation with peer service operators should consider different response times within geographic regions to ensure appropriate interventions are available in the event of an overdose. Only two peer reviewed studies have shown early effectiveness in reducing illicit drug mortality. 7,29,50 Continued evaluation of these services and data transparency on behalf of provincially run timer-based application services like the DORS and LifeGuard apps may help to counter current apprehension within the first responder community.

False alarm calls were also addressed within participant responses. Combined with extraordinarily high rates of first responder calls for opioid-related events, 2 the potential to burden an already overburdened community is high. Current research of one service, however, has shown limited numbers of false alarm calls and a positive cost-benefit ratio. 29,50,51 These figures fall far below those reported in other automatic dispatch services, such as fall alarms. 52 To our knowledge, there is no peer-reviewed literature describing the rates of false alarms in timer-based applications. Continuous monitoring is needed to ensure that these services are resources to respond to emergency calls adequately and do not contribute to significant additional stress on EMS professionals.

Lastly, participants discussed the potential strengths that peer support may offer on hotline-based services. Previous studies about connecting to in-person peer support have demonstrated improvements in patient engagement and knowledge translation. 53–56 Previous studies of application-based services have also demonstrated their capability to disseminate public health information. 36 Future research may help determine whether overdose response hotlines and apps demonstrate similar results and outcomes among PWUD populations. 56,57

### Current Awareness of Overdose Response Hotlines and Applications Should be Improved

As highlighted within a recent scoping review, Bolster and colleagues suggest that paramedic-attended overdose events are a unique and valuable opportunity to provide meaningful engagement with PWUD beyond an overdose response. 21 The results from our study suggest that paramedics demonstrated a willingness to engage with individuals beyond emergency response, providing clients, family members, and friends with resources that may help to reduce the harm associated with substance use. First responders have previously expressed their desire to provide quality care and education to PWUDs in need of resources and support. 30 Against the backdrop of high rates of overdose within a year since using EMS, 58 particularly when individuals are not transported to additional care resources, 59 these interactions are a critical window for providing resources and support for PWUD.

Interview participants noted that it was common that they would provide naloxone kits to individuals after an overdose event and, indeed, these have been previously considered as a public health messaging tool. 28 Previous studies note that individuals and family members who were offered and accepted naloxone kits were 2.47 and 5.6 times more likely to seek out substance use support than those who did not receive a kit, respectively. 60 The provision of naloxone kits in combination with education on ORT and the supports provided therein may help make additional strides in reducing the harms associated with illicit drug use. However, challenges may arise from interacting with individuals recovering from overdose or in naloxone-precipitated withdrawal due to the altered mental states associated with these conditions. 61

Lastly, interviewee perspectives were mixed regarding trained members of the public responding to overdoses within their communities. Concerns about community member safety, liability, training, critical incident stress and delays in emergency medical services were all raised. As previously mentioned, education for ORT users regarding responder safety would likely go a long way toward this goal; however, issues regarding potential aggression resulting from precipitated naloxone withdrawal would remain to be addressed. Training community responders regarding life-saving interventions, including naloxone administration, airway protection in conjunction with liability protections offered by the Good Samaritan Act, and safety concerns in the event of precipitated withdrawal, should also be provided. Critical incident-stress impacts should also be considered for individuals responding to overdoses through these services to minimize stressors and potential long-term mental health impacts from these events. In regard to delays in EMS, one ORT, Unity Philly, had previously used and studied this strategy for overdose response and noted that in 74 cases (59.5% lay-person intervention preceded interventions by emergency medical services by greater than five minutes. 62

## LIMITATIONS

When interpreting the findings contained within our study, a few limitations must be considered. Firstly, interviews were conducted in the province of Alberta and, thus, may not apply to the experiences of all EMS personnel across Canada or internationally. As the DORS has been a province-led initiative, awareness of overdose response hotlines and apps is likely higher than that seen in other jurisdictions. Additionally, the convenience and snowball sampling nature of our participant pool may have reduced the diversity of responses contained within the study. Efforts were made to recruit individuals from diverse geographic, occupational, and experience backgrounds to incorporate diverse perspectives of these individuals in their care for PWUD. The results of this study do not prove the effectiveness of this strategy in reducing the harms associated with illicit drug poisoning, and additional research should be conducted to determine measurable outcomes from integrating education and the provision of resources for emergency responses, particularly in the context of individuals refusing treatment. Lastly, due to issues with data collection, we were unable to collect the demographic data from every participant, and thus our results do not fully represent the ethnicities and years of service of our participant sample.

## CONCLUSION

Our findings reinforce the existence of continued pressures faced by first responders within the context of the opioid epidemic and highlight current reservations and suggestions regarding the implementation of overdose response hotlines and apps across the province of Alberta. Overall, first responders within the province of Alberta had a general awareness of the virtual harm reduction services available for PWUD; however, they had a limited understanding of their application and efficacy. Furthermore, while participants highlighted the various opportunities provided by these services, including more rapid response to overdoses, referrals to services, and connection to peer support, they expressed concerns about both personal and client safety during responses and false alarm calls. The results from our study demonstrate that discussing these services with clients is an acceptable strategy within the first responder community; however, additional steps should be taken to continue to evaluate these services and disseminate information amongst this population.

## Supplementary Information



## Figures and Tables

**Table 1:** Participant demographic data.

Pseudonym	Role	Previous awareness of overdose response hotlines and applications	Gender	Location	Years in service	Ethnicity
EMS 01	Firefighter/paramedic	Yes	Male	Urban	10	White
EMS 02	Community paramedic	Yes	Male	Urban	Not collected	Not collected
EMS 03	Community paramedic	Yes	Male	Urban	Not collected	Not collected
EMS 04	Firefighter/paramedic	No	Male	Urban	19	White
EMS 05	Fire Captain	No	Male	Urban	19	White
EMS 06	Primary care paramedic	No	Male	Urban	8	Asian
EMS 07	Advanced care paramedic	Yes	Female	Urban	Not collected	Not collected
EMS 08	Mobile integrated health and EMS paramedic	Yes	Male	Urban	17	White
EMS 09	Primary care paramedic	Yes	Non-binary	Rural	12	White
EMS10	Primary care paramedic	No	Female	Rural	4	White
EMS11	Primary care paramedic	Yes	Male	Rural	11	White
EMS12	Advanced care paramedic	Yes	Female	Rural	25	White
EMS13	Call evaluator	No	Female	Urban	1	White
EMS14	Emergency communications officer	Yes	Male	Urban	20	White
EMS15	Emergency communications officer	Yes	Male	Urban	1	White
EMS16	Emergency communications officer	No	Female	Urban	10	Indigenous
EMS17	Emergency communications officer	Yes	Female	Urban	3	White

*EMS*, emergency medical service.

**Table 2:** Summary of themes and sub-themes.

Theme	Subtheme	Key points
Current toll of overdose calls on emergency responders	Current overdose response frequency	Increase in frequency of overdoses Discussion of daily, monthly and annual approximate frequency metrics Geographical impact on overdose frequency
	Current management/response to overdose calls	Treatment disparities for patients/overdose calls Strained resources for responding to overdose calls Lack of protocols and procedures for managing/responding to overdoses Improving emergency response and questioning protocols Challenges of providing emergency medical instructions to impaired callers
	Impacts of responding on EMS/first responders	Emotional toll of dealing with the overdose crisis Cycle of frustration and exhaustion in responding to repeat overdose calls Increased danger and personal safety concerns in overdose calls Opportunities and impact for helping others in overdose calls
Acceptability of overdose response hotlines and applications	Automated overdose response apps and how they fit the needs of EMS/first responders	An important tool in harm reduction Early activation of emergency services is a strength of automated response apps Improved location accuracy for emergency response. Automated services are most appropriate for specific populations of people who use substances. Concerns about false alarm calls with automated apps compared to other medical alarm services
	Limitations of automated overdose response applications	Balancing harm reduction and enabling Possibility of false alarm calls Time-sensitivity and efficiency concerns Safety concerns for first responders Apprehension toward automated services as an emergency response tool Need to partner with further supports with automated overdose response applications Accessibility limitations for PWUD
	EMS/first responder perception of overdose response hotlines	Provide empathy and peer support in overdose response Provide personalized support and referral service for substance use Peer-to-peer services are targeted to specific populations Peer-to-peer services do not deter people from using substances
	Hotline overdose response services meet the needs of EMS/first responders	Enhanced information and context from peer-to-peer services Hotline services are more reliable/have fewer false alarms than automated services. Safety and liability concerns with peer-to-peer services Considerations of resource availability and time commitment Reliance on technology could pose safety issues.
	Impact of overdose response hotlines and applications on EMS/first responders	Overdose response hotlines and applications might lower the stress of overdose calls and result in better outcomes. Overdose response hotlines and applications could reduce the resource impact on the healthcare system by minimizing the number of false alarm calls. Impact and concerns about capacity and call volumes
	Perceived impact if overdose response hotlines and applications did not exist	If virtual harm reduction services didn’t exist, there would be more overdose calls and more strain on resources. There would be no impact as EMS/first responders have not seen these services used. If these services didn’t exist, there would be fewer avenues for compassionate care for PWUD.
Integration of overdose response hotlines and applications into communities and current workloads is feasible	Integrating automated applications into dispatch	Overdose response hotlines and applications could be integrated into dispatch protocols similarly to other alarm calls. Direct integration of overdose response hotlines and applications into emergency dispatch services can be helpful. Real-time feedback and follow-up services for EMS/first response could be conducted.
	Perspectives of lay responders to overdose response hotline and application dispatches	Trust and comfort in personal connections is key. Responding laypersons should be aware that they have been requested to respond and have the necessary training and resources to effectively respond to an overdose. Using friends and family as support persons is the most appropriate way to respond. Laypersons responding to overdoses could delay the appropriate response. Concerns about layperson response and associated trauma on them Concerns about the safety and effectiveness of layperson naloxone administration.
	Recommending overdose response hotlines and applications to patients	EMS/first responders would recommend these services to patients as a harm-reduction measure. EMS/first responders would recommend these services to certain patient populations only. There are barriers for promoting overdose response hotlines and applications in EMS dispatch.
	Appropriateness of discussing overdose response hotlines and applications with patients	Individuals are not likely to be receptive to information during post-resuscitation. Fatigue and burnout impacting EMS/first responder engagement in providing further services. Discussing ORT in post-resuscitation situations may sound patronizing. Discussing ORT in a post-resuscitation situation may not be appropriate for all patients. Challenges of meaningful interaction during opioid overdose response, including time constraints, complex cases and patient care prioritization
Current awareness of overdose response hotlines and applications should be improved	First responder awareness of overdose response hotlines and applications	Participants had heard of these services prior to the interview (n=12). Participants had not heard of these services prior to the interview (n=5). Overdose response hotlines and applications are technological forms of harm reduction. Overdose response hotlines and applications do provide privacy and anonymity in substance use support.
	First responders heard about overdose response hotlines and applications through a variety of means	Personal connection with an overdose response hotline and application advocate Presentation from an overdose response hotline and application advocate Advertisements for the services through posters or pamphlets Emails from management to notify staff of the services Reading about the services in articles Encountered overdose response hotline and application in their role as EMS/first response Through advertisements in naloxone kits Through TV advertisements
	Increasing awareness of overdose response hotlines and applications amongst first responders	Current awareness of overdose response hotlines and applications amongst first responders is lacking. Awareness of overdose response applications and hotlines is not necessarily needed for first responders. Education and training is needed for effective promotion and understanding by EMS/first responders. A detailed understanding of overdose response hotlines and applications is needed to increase buy-in from EMS/first response. Challenges in communicating and educating EMS/first responders about overdose response hotlines and applications are present. Effective communication channels for raising awareness of these services is needed among first responders.

*EMS*, emergency medical services; *ORT*, overdose response technologies; *PWUD*, people who use drugs.

## References

[1] Federal, Provincial, and Territorial Special Advisory Committee on the Epidemic of Opioid Overdoses (2023). Opioid- and stimulant-related harms in Canada.

[2] SAS® Visual Analytics Alberta substance use surveillance system.

[3] Khair S, Eastwood CA, Lu M (2022). Supervised consumption site enables cost savings by avoiding emergency services: a cost analysis study. Harm Reduct J.

[4] Bardwell G, Scheim A, Mitra S (2017). Assessing support for supervised injection services among community stakeholders in London, Canada. Int J Drug Policy.

[5] Hu K, Klinkenberg B, Gan WQ (2022). Spatial-temporal trends in the risk of illicit drug toxicity death in British Columbia. BMC Public Health.

[6] Loverock A, Marshall T, Viste D (2023). Electronic harm reduction interventions for drug overdose monitoring and prevention: A scoping review. Drug Alcohol Depend.

[7] Rioux W, Enns B, Ghosh SM (2023). Virtual overdose monitoring services/mobile overdose response services: Estimated number of potentially averted drug poisoning fatality events by various telephone and digital-based overdose prevention/harm reduction services in North America. Front Public Health.

[8] Rioux W, Marshall T, Ghosh SM (2023). Virtual overdose monitoring services and overdose prevention technologies: opportunities, limitations, and future directions. Int J Drug Policy.

[9] National Overdose Response Service (NORS) National overdose response service (NORS).

[10] Never Use Alone Inc Never use alone.

[11] The Brave App The Brave app.

[12] Lifeguard Digital Health Lifeguard digital health.

[13] Rioux W, Teare A, Rider N (2024). Preference for hotline versus mobile application/countdown-based mobile overdose response services: A qualitative study. Harm Reduct J.

[14] Tsang VWL, Papamihali K, Crabtree A (2021). Acceptability of technological solutions for overdose monitoring: Perspectives of people who use drugs. Subst Abuse.

[15] Tas B, Lawn W, Traykova EV (2023). A scoping review of mHealth technologies for opioid overdose prevention, detection and response. Drug Alcohol Rev.

[16] Marshall T, Viste D, Jones S (2023). Beliefs, attitudes and experiences of virtual overdose monitoring services from the perspectives of people who use substances in Canada: a qualitative study. Harm Reduct J.

[17] Perri M, Schmidt RA, Guta A (2022). COVID-19 and the opportunity for gender-responsive virtual and remote substance use treatment and harm reduction services. Int J Drug Policy.

[18] Perlmutter D, Wettemann C, Fockele CE (2023). “Another tool in the toolkit”—Perceptions, suggestions, and concerns of emergency service providers about the implementation of a supervised consumption site. Int J Drug Policy.

[19] McNeil R, Dilley LB, Guirguis-Younger M (2014). Impact of supervised drug consumption services on access to and engagement with care at a palliative and supportive care facility for people living with HIV/AIDS: a qualitative study. J Int AIDS Soc.

[20] Pollini RA, Paquette CE, Drvar T (2021). A qualitative assessment of discharge against medical advice among patients hospitalized for injection-related bacterial infections in West Virginia. Int J Drug Policy.

[21] Bolster J, Armour R, O’Toole M (2023). The paramedic role in caring for people who use illicit and controlled drugs: A scoping review. Paramedicine.

[22] Alberta (2024). Current provincial population estimates.

[23] Government of Canada SC (2022). Population growth in Canada’s rural areas, 2016 to 2021.

[24] Mertz E (2022). EMS crews have saved 18 patients using Alberta’s overdose response app.

[25] Proctor E, Silmere H, Raghavan R (2011). Outcomes for implementation research: conceptual distinctions, measurement challenges, and research agenda. Adm Policy Ment Health Ment Health Serv Res.

[26] Glaser B, Strauss A Discovery of Grounded Theory: Strategies for Qualitative Research.

[27] DORS App Dorsapp.

[28] Safi F, Rioux W, Rider N (2023). Feasibility and acceptability of inserts promoting virtual overdose monitoring services (VOMS) in naloxone kits: A qualitative study. Harm Reduct J.

[29] Matskiv G, Marshall T, Krieg O (2022). Virtual overdose monitoring services: a novel adjunctive harm reduction approach for addressing the overdose crisis.

[30] Witkowski K (2023). Lessons from the frontlines of the overdose crisis: exploring the impact of harm reduction policies on the roles and responsibilities of first responders. Public Pers Manag.

[31] Williams-Yuen J, Minaker G, Buxton J (2020). ‘You’re not just a medical professional’: exploring paramedic experiences of overdose response within Vancouver’s downtown eastside. PLos O ne.

[32] Pike E, Tillson M, Webster JM (2019). A mixed-methods assessment of the impact of the opioid epidemic on first responder burnout. Drug Alcohol Depend.

[33] Sable P, Tang F, Swab JA (2023). EMS workers on the frontline of the opioid epidemic: effects of sleep and social support on depression. Int J Emerg Serv.

[34] Antony J, Brar R, Khan PA (2020). Interventions for the prevention and management of occupational stress injury in first responders: a rapid overview of reviews. Syst Rev.

[35] Tay Wee Teck J, Oteo A, Baldacchino A (2023). Rapid opioid overdose response system technologies. Curr Opin Psychiatry.

[36] Oteo A, Daneshvar H, Baldacchino A (2023). Overdose alert and response technologies: state-of-the-art review. J Med Internet Res.

[37] Seo B, Rider N, Rioux W (2024). Understanding the barriers and facilitators to implementing and sustaining mobile overdose response services from the perspective of Canadian key interest groups: a qualitative study. Harm Reduct J.

[38] Bennett AS, Elliott L (2021). Naloxone’s role in the national opioid crisis—past struggles, current efforts, and future opportunities. Transl Res.

[39] Elliott L, Bennett AS, Wolfson-Stofko B (2019). Life after opioid-involved overdose: Survivor narratives and their implications for ER/ED interventions. Addiction.

[40] Wight H, Ratcliffe JH (2024). Aiding or enabling? Officer perspectives on harm reduction and support services in an open-air drug market. Polic Soc.

[41] Bessen S, Metcalf SA, Saunders EC (2019). Barriers to naloxone use and acceptance among opioid users, first responders, and emergency department providers in New Hampshire, USA. Int J Drug Policy.

[42] Knaak S, Christie R, Mercer S (2019). Harm reduction, stigma and the problem of low compassion satisfaction. J Ment Health Addict Nurs.

[43] Saunders E, Metcalf SA, Walsh O (2019). “You can see those concentric rings going out”: emergency personnel’s experiences treating overdose and perspectives on policy-level responses to the opioid crisis in New Hampshire. Drug Alcohol Depend.

[44] Ritter A, Cameron J (2006). A review of the efficacy and effectiveness of harm reduction strategies for alcohol, tobacco and illicit drugs. Drug Alcohol Rev.

[45] van Boekel LC, Brouwers EPM, van Weeghel J (2013). Stigma among health professionals towards patients with substance use disorders and its consequences for healthcare delivery: systematic review. Drug Alcohol Depend.

[46] Xavier J, Greer A, Crabtree A (2022). Police officers’ perceptions of their role at overdose events: a qualitative study. Drugs Educ Prev Policy.

[47] Kahn LS, Wozniak M, Vest BM (2022). “Narcan encounters:” overdose and naloxone rescue experiences among people who use opioids. Subst Abuse.

[48] Ritchie K, Ghosh SM (2022). Determining the feasibility for an overdose prevention line to support substance users who use alone. Harm Reduct J.

[49] Mell HK, Mumma SN, Hiestand B (2017). Emergency medical services response times in rural, suburban, and urban areas. JAMA Surg.

[50] Viste D, Rioux W, Cristall N (2023). Association of drug overdoses and user characteristics of Canada’s national mobile/virtual overdose response hotline: the national overdose response service (NORS). BMC Public Health.

[51] Rioux W, Enns B, Jackson J (2023). A cost benefit analysis of a virtual overdose monitoring service/mobile overdose response service: The national overdose response service. Subst Abuse Treat Prev Policy.

[52] Johnston K, Worley A, Grimmer-Somers K (2010). Personal alarm use to call the ambulance after a fall in older people: Characteristics of clients and falls. Australas J Paramed.

[53] Collins D, Alla J, Nicolaidis C (2019). “If it wasn’t for him, I wouldn’t have talked to them”: Qualitative study of addiction peer mentorship in the hospital. J Gen Intern Med.

[54] Bassuk EL, Hanson J, Greene RN (2016). Peer-delivered recovery support services for addictions in the United States: a systematic review. J Subst Abuse Treat.

[55] Ramdin C, Guo M, Fabricant S (2022). The impact of a peer-navigator program on naloxone distribution and buprenorphine utilization in the emergency department. Subst Use Misuse.

[56] Rider N, Safi F, Marshall T (2023). Investigating uses of peer-operated virtual overdose monitoring services (VOMS) beyond overdose response: A qualitative study. Am J Drug Alcohol Abuse.

[57] Viste D, Rioux W, Rider N (2024). Characteristics and risk of adverse mental health events amongst users of the national overdose response service (NORS) telephone hotline. Int J Ment Health Addict.

[58] Barefoot EH, Cyr JM, Brice JH (2021). Opportunities for emergency medical services intervention to prevent opioid overdose mortality. Prehosp Emerg Care.

[59] Zozula A, Neth MR, Hogan AN (2022). Non-transport after prehospital naloxone administration is associated with higher risk of subsequent non-fatal overdose. Prehosp Emerg Care.

[60] Scharf BM, Sabat DJ, Brothers JM (2021). Best practices for a novel EMS-based naloxone leave behind program. Prehosp Emerg Care.

[61] Khan GK, Harvey L, Johnson S (2022). Integration of a community-based harm reduction program into a safety net hospital: a qualitative study. Harm Reduct J.

[62] Schwartz DG, Ataiants J, Roth A (2020). Layperson reversal of opioid overdose supported by smartphone alert: A prospective observational cohort study. EClinicalMedicine.

