# Supplementary material for: “Oh, Another Overdose, for the Love of Pete”: First Responder Perspectives on Overdose Response Technology
Source: West J Emerg Med. 2025 Feb 12;26(3):588–99. doi: 10.5811/westjem.18471 (PMC12208050; doi:10.5811/westjem.18471)
Supplement: Supplementary file 1 [file wjem-26-588-s001.docx]

Virtual Supervised Consumption Services Evaluation

# Health Professionals/Emergency Services Interview information form

**Why do you want to talk to me?**

We would like to talk to you about something called virtual supervised consumption services. These services can help keep people safe when they use drugs and can be especially helpful for people who use alone. When individuals use some drugs, especially opioids, there can be a risk of fatal overdose.

Virtual supervised consumption services can be a telephone service or a smartphone app. Each of these methods monitor people who are using substances and activate emergency services or an emergency response plan if the person using becomes unresponsive.

We would like to hear from health professionals and emergency services professionals that provide health and emergency care to the people these services are trying to help to discuss the feasibility of the services being integrated into acute care

**What are you asking me to do?**

We would like you to talk with us for 30 - 45 minutes on the telephone. Some things we would like to talk to you about are what you like and don’t like about virtual supervised consumption services, and also what you think should be changed to help people who use or want to use alone.

You will talk to a trained evaluator from a contracted evaluation consulting firm called [Three Hive Consulting.](http://www.threehive.ca/) The conversation can be scheduled by clicking the Calendly link provided in the email. [Calendy](https://calendly.com/d/cgr-qr3-rgb/virtual-supervised-consumption-services-interview) is an automated scheduling platform that makes it easier for us to book a time with you. However, if you prefer you can call or email Stephanie Jones ([stephanie@threehive.ca](mailto:stephanie@threehive.ca) or 250.918.5351) to book a time.

**Are there risks to me if I talk to you?**

Talking about some things in your life could bring out uncomfortable feelings. You do not have to answer any questions or talk about anything you do not want to talk about. It is up to you. You can change your mind about taking part later. All you have to do is tell us you want to stop.

**Do you record the conversation?**

We would like to record what we talk about to make sure we get the right information; however, if you decide not to have what we talk about recorded that is okay. If you are okay with us recording what we talk about you should know it will be typed into a document and your (or any other) names will be removed. The recording will be erased from the recorder after the document is typed.

**Who will see or hear the information you collect?**

We will do everything we can to keep your identity and the information we talk about private. Only specific people from the research team and contracted evaluation firm (Three Hive) will have access to the name and other personal information provided, but no identifiable information will be included in any reports. As previously mentioned, you do not have to share your real name if you don’t want to. We may use a quote you say in our reporting; however, we will only use quotes where you cannot be identified. Once the report is written we will throw away all of this information.

**How does me talking to you help?**

You may not benefit from it personally. However, we might find out different things talking to you that we would not have known by just talking to people who design and implement the services. The information you tell us could help other people who use alone and think the services might help.

**Do I have to talk to you?**

You do not have to talk to us. It’s up to you. If you want to talk to us, you can change your mind at any time. All you have to do is tell us.

**Who do I call if I have more questions?**

Dr. S. Monty Ghosh, Research Lead (403-367-5000)

Shelby Corley, Three Hive Consulting Evaluation Lead (780-399-1273)

Virtual Supervised Consumption Services Evaluation

# Health Professionals/Emergency Services Interview Script and guide

**Introduction**

Thank you for agreeing to talk with me today. We would like to talk to you about something called virtual supervised consumption services. These services can help keep people safe when they use drugs and can be especially helpful for people who use alone. As you know, when individuals use some drugs, especially opioids, there can be a risk of fatal overdose. Virtual supervised consumption services can be a telephone service or a smartphone app. Each of these methods monitor people who are using substances and activate emergency services or an emergency response plan if the person using becomes unresponsive.

We would like to hear from health professionals and emergency services professionals that provide health and emergency care to the people these services are trying to help to learn if they can be used to help other people who choose to use substances alone. Some things we would like to talk to you about are what you like and don’t like about the services, as well as what you think the impacts of the services are and what should be changed to help people who use or want to use alone. There are no right or wrong answers to my questions. We are hoping the conversation won’t take more than 45 minutes.

Please know that I do not have a personal interest in virtual supervised consumption services in general, so please feel free to speak openly and honestly. Everything you say is voluntary and will be kept confidential to the extent explained earlier.

Do you have any questions? Is it okay to proceed?

**<If yes,** proceed to age screening question>

<**If no,** ask if and what information needs to be clarified. Clarify and again ask if it is okay to proceed>

**Age Verification**

Before we begin, are you currently 18 years of age or older?

**<If yes,** proceed to first question>

<**If no,** thank the person for their time and let them know that the person must be 18 years of age to participate>

| **EMS & First Responder Questions Around Remote/Digital Overdose Monitoring**  **Alberta, Canada (2022)** | | |
| --- | --- | --- |
| **Topic** | **Question type** | |
|  | ***Qualitative*** |  |
| 1. Burden of overdose calls | Over the past year, how have overdose-related calls impacted you personally?  Probe: Can you further describe any positive and negative experiences?  Probe: How have overdose calls impacted your unit and/or the EMS/Fire system at large? |  |
| 1. Supervised consumption services | Supervised consumption services are physical sites where people who use drugs can use drugs under the supervision of trained staff who can respond to any adverse events such as an overdose.  Please describe, in as much detail as possible, how do you feel about this type of service?  Probe: Do you feel this service may be helpful or harmful? i.e., what are the strengths and limitations of this service from your point of view?  Probe: How do you feel a service like this might impact your work or your unit? |  |
| 1. Automated/digital overdose monitoring services | Please describe, in as much detail as possible, how do you feel about this type of service?  Probe: Do you feel this service may be helpful or harmful? i.e., what are the strengths and limitations of this service from your point of view?  Probe: How do you feel a service like this might impact your work or your unit? |  |
| 1. Virtual overdose monitoring services | Virtual overdose monitoring services connect a substance user to a line operator, which can facilitate a premade emergency response plan.  Describe, in as much detail as possible, how do you feel about this type of service? i.e., what are the strengths and limitations of this service from your point of view?  Probe: Do you feel this service may be helpful or harmful?  Probe: Do have any concerns about “false alarm” calls? How might this impact resources? Do you experience this often?  Probe: How do you feel a service like this might impact your work or your unit?  Probe: how do you feel about laypersons (e.g., friend, family members) responding to overdoses? |  |
| 1. Policy/Practice | What do you think needs to be done to better address the overdose crisis?  Probe – what policies/practices would you like to see EMS/fire change in help address the overdose crisis?  Probe – what specific things would you like to see changed when responding to an overdose call? (For example, who responds to the call, what the protocols and procedures are?)  Probe – what would you like to see changed to improve your experience as a first responder? |  |
| 1. Other/exploratory | Do you have any other questions, comments, or concerns about the overdose crisis, SCS or overdose monitoring services? |  |
